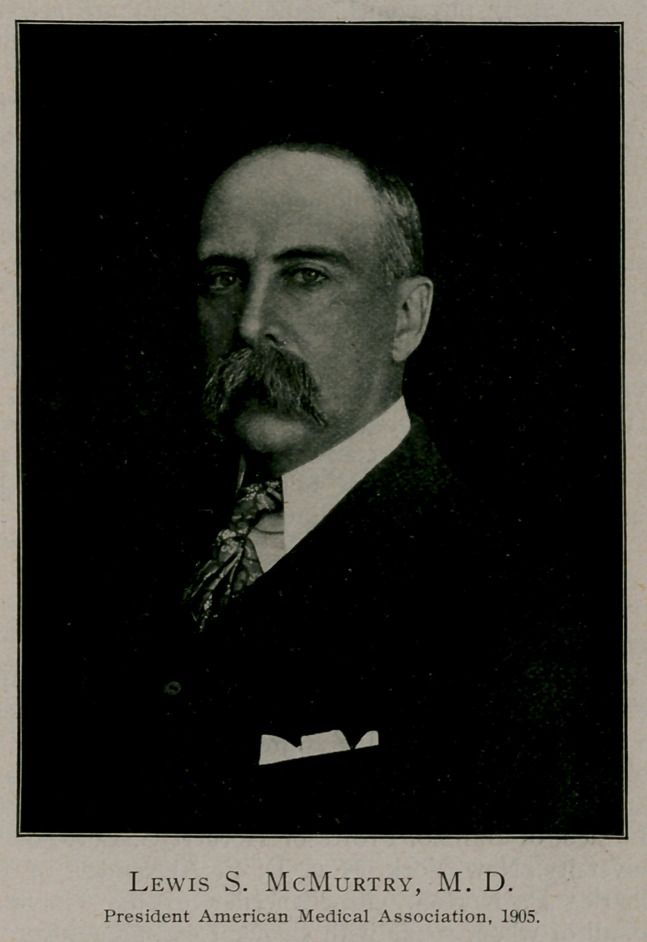# The American Medical Association at Portland

**Published:** 1905-07

**Authors:** 


### The American Medical Association at Portland.

THE fifty-sixth annual meeting of the American Medical Association is scheduled to be held at Portland, Ore., July 11-14, 1905. At this writing the indications are that the attendance will be large and the work of the association of a much higher order than generally has been the case with meetings on the Pacific coast.

LEWIS S. MCMURTRY, M. D.

President American Medical Association, 1905.

There are several reasons why this prognostication seems justifiable, among which may be mentioned first, speed and comfort of trains; second, low rates and convenient schedules; third, the improved organisation of the association; and fourth, the additional attraction lent by the Lewis and Clarke exposition. This latter adds a novelty to the occasion never before experienced at an association meeting.

To visit the Pacific slope becomes at once a duty and a pleasure to every American citizen residing east of the Rocky mountains who can afford the time and money. For the young it is a part of the education scheme that should enter into every curriculum; and to older persons it furnishes an accomplishment that should not be neglected. The opportunity presented at this time is an unusual one for the fulfilment of these objects that play such important parts in the career of every useful citizen.

Already, many physicians, some of whom are accompanied by members of their families, are on the way to the meetings that are scheduled to take place before or during the gathering of the great association itself. Among these are President McMurtry and his daughter, of Louisville, Dr. A. Vander Veer and wife, of Albany, Dr. George Ben Johnston, of Richmond, Dr. Roswell Park, of Buffalo, and others from Boston, New York, Philadelphia, Chicago and other cities, who go to attend the meeting of the American Surgical Association to be held at San Francisco, July 5, 6, and 7, under the presidency of Dr. George Ben Johnston, of Richmond.

The last date practicable upon which to start from Chicago, in order to reach the association meeting at Portland, is July 6; hence, those who contemplate being present on that occasion will find it necessary to make transportation arrangements as soon as this edition of the journal falls into their hands. Further delay in this matter will prove impracticable.

---

University extension has received substantial impetus of late in one or two important directions. President Thwing, of Western Reserve University, addressed a representative gathering of college men and friends of the Buffalo institution at the university club on the evening of May 27, 1905. Much enthusiasm was manifested and the distinguished speaker was followed by Mr. J. N. Larned, of Buffalo, Professor Herbert G. Lord, of Columbia University, New York, Hon. D. S. Alexander, of Buffalo, and Charles P. Norton, vice-chancellor of the University of Buffalo, all of whom spoke with eloquent encouragement.

Subscriptions to a fund have been offered as follows: Mrs. E. C. Sprague, $5,000; "Citizen," $2,500; Arthur Detmers, $500; Walter L. Brown, $100. This is a modest beginning and should stimulate activity on the part of citizens of Buffalo who have ample means. We should suppose that civic pride would lead to contributions covering the amount needed within a comparatively short time.

Now is the time to lay in a supply of tetanus antitoxin for use on the "glorious Fourth."

The foregoing from a prominent daily newspaper is an unpleasant reminder of the tragedies that have been enacted heretofore in the name of the celebration of our national holiday.

If, however, the ordinance adopted in Buffalo relating to the sale of explosives is adequately enforced, as the superintendent of police promises it shall be, there will be no need of the remedy indicated. The following provisions of the ordinance, adopted at the instigation of the Medical Society of the County of Erie, should be borne in mind:

It prohibits the sale or giving away or having in their possession for use of any kind of fireworks containing dynamite, giant powder, nitroglycerine, dualin or other explosive more powerful than ordinary black gunpowder.

It prohibits the sale or giving away of "any giant firecracker, or any other firecracker that is likely to maim or injure any person by the explosion thereof, except Chinese firecrackers not exceeding five inches in length."

It forbids the sale or distribution of the following specified articles:

Toy revolvers, toy pistols or toy cannon of any kind in which powder can be exploded. Blank cartridge pistols, toy cartridge pistols or toy revolvers. Repeating or bombjack marbles. Kango clubs. Car-track torpedoes. Vesuvius torpedoes. Torpedo canes or ammunition for same.

The ordinance further says: "No person shall place any torpedo of any description upon the street-car tracks or upon the public streets of the city."

Its final provision forbids the sale of any kind of fireworks to children under fifteen years old.

And for a violation of any of the provisions of this ordinance, it prescribes a fine of $25 for each offense.

---

THE state cancer laboratory, located at Buffalo, receives the usual appropriation of $15,000 for the next fiscal year. This amount, contained in the supply bill, received the governor's approval, and will enable the director, Dr. Roswell Park, to proceed with the investigations relating to the causes of cancer.

---

THE Baly medal has been awarded to Professor Pawloff, of Saint Petersburg. This medal is given every alternate year on the recommendation of the president and council of the Royal College of Physicians of London for distinguished work in the science of physiology, especially during the two years immediately preceding the award. The Bisset Hawkins gold medal for 1905, given triennially for work deserving special recognition as advancing sanitary science or promoting public health, has been awarded to Sir Patrick Manson.

---

THE Jackson Health Resort, at Dansville, N. Y., held the annual commencement exercises of the nurses' class Thursday evening,

June 15, 1905, at which ten nurses were graduated. Dr. Matthew D. Mann, of Buffalo, delivered the address.

---

APOLLINARIS, in spite of all rivals, still retains its justly established title,—"The Queen of Table Waters." It is alike grateful to the invalid and refreshing to the healthy person. Physicians not only prescribe it, but drink it themselves. As a health restoring and maintaining beverage it is unrivalled among the potable waters of the world.

## Figures and Tables

**Figure f1:**